# Sugar Reduction in Dairy Food: An Overview with Flavoured Milk as an Example

**DOI:** 10.3390/foods9101400

**Published:** 2020-10-02

**Authors:** Dipendra Kumar Mahato, Russell Keast, Djin Gie Liem, Catherine Georgina Russell, Sara Cicerale, Shirani Gamlath

**Affiliations:** CASS Food Research Centre, School of Exercise and Nutrition Sciences, Deakin University, Burwood, VIC 3125, Australia; russell.keast@deakin.edu.au (R.K.); gie.liem@deakin.edu.au (D.G.L.); georgie.russell@deakin.edu.au (C.G.R.); sara.cicerale@deakin.edu.au (S.C.); shirani.gamlath@deakin.edu.au (S.G.)

**Keywords:** sugar reduction strategies, flavoured milk, sweeteners, stevia, monk fruit

## Abstract

Owing to the public health concern associated with the consumption of added sugar, the World Health Organization recommends cutting down sugar in processed foods. Furthermore, due to the growing concern of increased calorie intake from added sugar in sweetened dairy foods, the present review provides an overview of different types and functions of sugar, various sugar reduction strategies, and current trends in the use of sweeteners for sugar reduction in dairy food, taking flavoured milk as a central theme where possible to explore the aforementioned aspects. The strength and uniqueness of this review are that it brings together all the information on the available types of sugar and sugar reduction strategies and explores the current trends that could be applied for reducing sugar in dairy foods without much impact on consumer acceptance. Among different strategies for sugar reduction, the use of natural non-nutritive sweeteners (NNSs), has received much attention due to consumer demand for natural ingredients. Sweetness imparted by sugar can be replaced by natural NNSs, however, sugar provides more than just sweetness to flavoured milk. Sugar reduction involves multiple technical challenges to maintain the sensory properties of the product, as well as to maintain consumer acceptance. Because no single sugar has a sensory profile that matches sucrose, the use of two or more natural NNSs could be an option for food industries to reduce sugar using a holistic approach rather than a single sugar reduction strategy. Therefore, achieving even a small sugar reduction can significantly improve the diet and health of an individual.

## 1. Introduction

The consumption of excessive free or added sugar contributes to total energy intake, and, possibly, to increased body weight [1], the occurrence of obesity [2,3], and associated chronic diseases such as type 2 diabetes [4,5]. Flavoured milk is used to promote milk intake to meet the recommended dietary allowances (RDA) for vitamin D and calcium [4]. Milk appears to be the principal dairy product consumed by children worldwide. It is estimated that between 60–80% of American children’s dairy product consumption is comprised of fluid milk [6]. Furthermore, 68% of all milk available to children in schools in the USA is flavoured, of which the majority is chocolate milk [7]. However, the regular consumption of sweetened flavoured milk has been reported to increase energy intake more than 10% as compared with non-consumers [8,9,10]. The increased energy intake is further linked to the occurrence of overweight, obesity [1,2,3], and type 2 diabetes [4,5]. 

The World Health Organization (WHO) recommends less than 10% of total energy intake from free sugars per day in both adults and children (strong recommendation). A further reduction to below 5% is a conditional recommendation [11]. These guidelines have been considered by Public Health England (PHE), which recommends a 20% sugar reduction in processed foods and beverages by 2020 [12]. A well-tested model of an epidemiological triad (hosts, vectors, and environments) provides a framework to address such public health concerns [13,14]. The vectors rule of this model suggests ”small changes × large volumes = significant population benefits”. Therefore, even a small reduction can significantly benefit a larger population in the long term. 

Hence, the main focus of this review is to provide an overview of different types and functions of sugar present in processed foods and beverages, current trends in the use of sweeteners, as well as various sugar reduction strategies that could be applied for sugar reduction in milk-based products without a significant impact on consumers’ sensory acceptance.

## 2. Types of Sweetener 

Sweeteners can be categorized into nutritive sweeteners (NSs) and non-nutritive sweeteners (NNSs) based on their nutritive value and sweetness potency (i.e., relative sweetness equivalent to sucrose).

### 2.1. Nutritive Sweetener (NS)

The NSs include sugars such as sucrose, fructose, and lactose, as well high-fructose corn syrup (HFCS), trehalose, and polyols (erythritol, isomaltitol, lactitol, maltitol, sorbitol, mannitol, and xylitol) [15,16]. NSs have various advantages when added to foods and beverages (Table 1), however, they provide calorie contribution. For these reasons, they are not preferred for sugar reduction strategies where calorie reduction is important.

### 2.2. Non-Nutritive Sweeteners (NNSs)

Non-nutritive (intensive) sweeteners (NNSs) are food additives with high sweetness potency. They are usually added in low amounts, and therefore their calorie contribution is almost negligible and are preferred for use where calorie reduction is desired (Table 2) [15]. NS and NNS can both be either natural or artificial [17,18,19]. Natural sweeteners are intrinsic to a food substance or commonly occur in nature, e.g., stevia and monk fruit [20], while artificial sweeteners are not found in nature but are synthesized from an existing natural source. The first artificial sweetener approved by the FDA was Saccharin in 1958, while Advantame was the most recent one approved by the FDA in 2014. Similarly, the first natural NNS approved for use by the FDA in 2009 was steviol glycosides with rebaudioside A as the main component. Furthermore, the physiological effects relating to NNSs and NSs vary greatly. NSs play more of a role in the regulation of hormonal secretion and brain activation to control appetite as compared with NNSs [21]. Considering this evidence, NNSs may serve as a good substitute for sugar reduction strategies.

## 3. Functions of Sugar

Sugar (sucrose) performs different functions in beverages. It provides sweetness and also helps to balance other tastes such as sour, salty, and spicy in less sweet products [51]. An important reason for using sugar in beverages is because it is a cheap and an efficient way to increase the liking and acceptance of foods.

### 3.1. Sweetness

The primary role of sugar is to provide sweetness to foods. The sweetness profile of sugar depends upon the quality and type of sugar [52]. Sweet is one of the four basic tastes [53]. The chemical tastants for sweetness bind to taste receptor cells (TRC) in the oral cavity and activate the intracellular signaling elements [54,55]. The receptors initiate a signal of the information to the taste processing regions of the brain through afferent nerve fibres [56]. In addition, the sweet taste signaling mechanism also operates in the gastrointestinal (GI) tract [57,58,59,60]. Therefore, a taste reception throughout the alimentary canal may influence satiety and regulate energy intake in an individual [21,61]. 

In terms of perception, there are four dimensions of taste, i.e., quality, intensity, temporal, and spatial patterns [62]. The quality attribute describes the sensations of taste compounds into four basic tastes, i.e., sweet, sour, salty, and bitter [53]. The intensity of the taste compounds is influenced by their concentration. The temporal aspect relates to the time duration of the intensities perceived, while the spatial attribute denotes the location on the tongue and oral cavity for taste sensations [62]. The sweetness perceived from sucrose varies from other types of sweeteners based on these four dimensions which have the potential to influence consumer acceptance. Furthermore, the sweetness perception can be triggered by the addition of vanilla, caramel, or fruity aromas [63,64,65].

### 3.2. Flavour

Milk naturally has lactose that participates in numerous Maillard reactions. A Maillard reaction occurs when amino groups interact with sugars [66]. The reaction leads to the formation of brown nitrogenous polymers or melanoidins along with other compounds having specific flavours [67]. The flavour compounds mainly include aldehydes, imines, acetal, diacetyl, furfural, and hydroxymethylfurfural (HMF). These are formed due to the breakdown of sugars, amino acids, and other intermediate compounds during Maillard reactions. The reaction is enhanced at a higher temperature and pH. In foods containing sugar, the Maillard reaction occurs simultaneously with caramelisation. The reaction occurs during pasteurisation and UHT treatment of milk [51]. The flavour compounds produced in milk by Maillard reactions include Strecker aldehydes, S- and N-containing compounds, maltol, and diacetyl [68,69]. The flavour in flavoured milk may occur due to Maillard reactions during the heat treatment. Therefore, sugar reduction in flavoured milk may reduce Maillard reactions which may further impact consumer acceptance. Having said that, however, this connection has not been established with flavoured milk.

### 3.3. Mouthfeel

Sugar is utilized to provide the desired mouthfeel in beverages. Sucrose contains hydroxyl (–OH) groups that interact with water in beverages to form hydrogen bonds. This increases the viscosity or bulk of the product and provides a proper “mouthfeel” if added in sufficient quantity [70]. A lower sugar concentration from 5% to 10% is used for sweetening of beverages which increases the viscosity and gives the perception of “mouthfeel” without the sensation of thickness. However, when sugar is used at higher concentrations from 60% to 75%, as in the case of syrups, the thickening effect can be observed easily. The interaction of sugar with water alters the behaviour of hydrophilic compounds such as proteins, starches, and hydrocolloids [70]. Sugar is usually mixed with hydrocolloids (e.g., gums and pectin) before adding to a liquid medium such as fruit juice in order that hydrocolloid molecules can hydrate properly and provide the desired mouthfeel. Similar to sucrose, fructose and lactose are ideal for providing mouthfeel of a product. Due to their higher solubility, they provide the desired mouthfeel in food and drinks [51,71]. In addition, mono- and disaccharides, the higher molecular weight oligosaccharides also provide increased viscosity and ultimately improve the body and mouthfeel of beverages [72,73]. Therefore, sugar reduction strategies with the aid of high potency sweeteners often require other ingredients such as starch and gums to compensate for the bulk deceased by a reduced portion of sugar.

### 3.4. Food Safety 

Sugar has an important role in food industries because of its significance in processing and shelf-life control [74,75]. The unique preservative property of sugar (sucrose), in addition to other functionalities, make it an essential nutrient in the world diet for ensuring food safety. Industrial sugar is usually obtained by processing of sugar cane and sugar beet. The reduction of sugar alters (increases) the water activity (a_w_) level in sweetened milk-based beverages that further creates a favourable environment for lipid oxidation, non-enzymatic browning (Maillard reaction), the growth of microorganisms, and enzyme activity that pose safety and stability issues to the product [76].

The defects of milk quality include excessive acidity, microbiological and mechanical impurities, and changed sensory qualities, i.e., taste, smell and colour. The degree of intensity of these defects determines the speed of the deterioration of milk quality and is closely related to the number of bacteria in the milk and its storage temperature. The consequences of bacterial growth in milk can be seen on its physicochemical properties [77,78]. The major changes are observed for colour, pH, acidity, viscosity, and development of off-flavours. The off-flavours develop due to fat hydrolysis [79], while changes in pH, acidity, and viscosity occur due to fermentation of lactose into lactic acid [80].

Milk provides a favourable environment for the growth of many microorganisms such as mesophilic and psychrotrophic bacteria. Milk is pasteurised at 72 °C for 15 s to check their growth. However, as the pasteurised milk is stored under refrigerated temperatures, psychrotrophic spore-formers, especially the *Bacillus* spp. can predominate [81]. Sometimes, even post-pasteurisation, the shelf-life is at risk due to the growth of endospore-forming and heat-resistant bacteria [82,83]. The psychrotrophic bacteria are thermolabile, and thus inactivated during pasteurisation, however, the enzyme (lipase) produced by these bacteria is heat resistant. The enzyme causes free fatty acids (FFAs) to release through lipid hydrolysis that develops the rancid flavour in milk [84,85]. Therefore, the inactivation of these enzymes is crucial to guarantee the safety and quality of milk [86]. Furthermore, the safety and quality of milk need attention when reducing sugar in sweetened dairy products.

## 4. Chemistry of Sweet Taste

Sweet taste provides a cue for calorie-rich food which innately attracts animals and humans. However, this attraction to sweetness poses a significant concern for human health [87]. Sugar replacement is a challenge for the food industry, and knowledge of structure-taste relationships can provide insights into the chemical space associated with a sweet taste [88]. All sweet-tasting compounds are detected by a heterodimer composed of two class C G protein-coupled receptors (C GPCRs), T1R2 and T1R3 subunits, which are expressed at the surface of the taste buds [89,90]. Some additional pathways such as glucose transporters and ATP-gated K+ channels have also been proposed for sweet taste recognition [91,92].

Sweet taste receptors can recognize low to high molecular weight, and artificial or natural compounds [93]. In addition, allosteric modulators of the sweet taste receptor have been reported, as observed for other class C GPCRs [94,95]. For example, positive allosteric modulators (PAMs) amplify the receptor response effect as evoked by sweet compounds. Hence, this may be utilized to reduce sugar intake while still maintaining the desired sweetness level [17,96,97]. Molecular modelling plays a significant role in the characterization of the binding modes of different modulators for the sweet taste receptor. The PAMs bind at an allosteric site which is different from the orthosteric site, the canonical site of T1R2 and T1R3 agonists [95]. Furthermore, negative allosteric modulators (NAMs) such as lactisole and gymnemic acids, have been predicted to have been located at a different binding site within the T1R3 TM domain [98,99]. In addition, molecular modelling has also revealed the binding modes of sweet compounds into the orthosteric binding site [100]. It has been predicted that the volume of T1R2 and T1R3 orthosteric binding pockets are big enough to bind small and large sweeteners in an open form [101].

In addition, several machine learning methods based on physicochemical properties and fingerprints of molecules [87] have been developed to predict the sweetness of molecules [102,103]. Furthermore, it is interesting to note similarities between sweet detection in the oral cavity by sweet oral taste receptor cells (TRC) and in the gastrointestinal tract (GIT) by the gastrointestinal sweet TRC [104,105,106,107,108]. The taste perception is initiated in the oral cavity while satiety hormones’ release is initiated in the GIT [90]. The existence of an identical nutrient-sensing mechanism in the oral cavity and GIT seems reasonable since both are part of the alimentary canal and accountable for the identification of nutrients and non-nutrients in foods [90], along with regulating the sweet taste perception of various sweeteners [62].

## 5. Sugar Reduction Strategies

To achieve sugar reduction targets, several strategies could be implemented such as improving dietary behaviour, minimizing the marketing of high sugar foods, and shifting consumer purchase behaviour towards low and no added sugar products, reformulating products with lower concentrations of sugar, and imposing sugar excise tax are the major strategies [109]. Among these, the effective way to attain sugar reduction would be to decrease the added sugar content of the processed products [110]. Product reformulation with partial substitution of sugar using suitable sweeteners is the most preferred method for sugar reduction in foods and beverages [111]. However, caution should be taken while reducing sugar, the reduction should be carried out in such a way that it meets the sensory expectations of consumers, if not they would be expected to reject the products even if the products are better for health [112]. 

Among the various methods for sugar reduction, the major ones are: (i) Lactose Hydrolysis, (ii) Ultra-/Nanofiltration, (iii) Product Reformulation by Partial or Total Replacement with Sweeteners, (iv) Gradual Reduction of Sugar, (v) Use of Multisensory Interactions, and (vi) Heterogeneous Distribution [32,62]. These methods are briefly described in the following subheadings and their feasibilities and applications in food and beverages are summarized in Table 3. 

### 5.1. Lactose Hydrolysis

Lactose hydrolysis can be utilized as a method for sugar reduction in dairy foods and beverages. Currently, enzymatic lactose hydrolysis has been used to produce lactose-reduced milk [113,114]. Lactose hydrolysis can be achieved either by adding β-galactosidase to pasteurised milk and storing the mixture for around 10–12 h, at 35–45 °C, and then applying further heat to de-activate the enzyme or by adding lactase to UHT milk before packaging where lactose is subsequently hydrolysed into glucose and galactose over a few days [115]. Lactose hydrolysis in milk causes approximately 70% of the lactose breakdown and increases the sweetness equivalent to two per cent of added sugar [115,116,117], therefore, increasing sweetness as compared with regular milk [117,118]. Additionally, a study investigated the application of lactose hydrolysis to naturally sweeten chocolate milk and revealed that the hydrolysis of lactose could not reach the sweetness desired for the chocolate milk, presumably due to the cocoa present in chocolate milk which had some bitterness [119]. Li, Lopetcharat, Qiu, and Drake [119] further tried adding lactose directly through the application of permeate followed by hydrolysis, but the permeate powder resulted in an intense salty taste due to the presence of minerals, and ultimately the approach failed to sweeten chocolate milk [119]. However, lactose hydrolysis could apply to other flavoured milks such as vanilla or strawberry milk. Furthermore, lactose hydrolysis has been applied as a means of sugar reduction in yoghurt [120,121,122] where the consumers could not detect a difference between yoghurt containing 4 g of sucrose/100 g of yoghurt and the lactose-hydrolysed yoghurt with less than 2–3 g of added sucrose/100 g of yoghurt. In addition to this, a 25% reduction in sugar was achieved by lactose hydrolysis in ice-cream [123].

### 5.2. Ultra- and Nanofiltration

The single-use of enzymes (lactase/β-galactosidase) for lactose hydrolysis is an expensive process [139]. Therefore, the membrane recovery system such as ultra- and nanofiltration are extremely helpful for the recovery and reuse of the enzymes. Ultrafiltration is a pressure-driven process that removes lactose from milk, and thus can be used as a sugar reduction technique [140]. The high molecular weight compounds such as fat and protein are retained by the ultrafilter membrane, while the low molecular weight compounds (lactose, minerals, vitamins and water) are able to pass through the membrane. Then, water is added to the suspended solids to obtain lactose-free milk. It is not as sweet as lactose-hydrolysed milk, therefore a NNS can be added to gain the desired sweetness [114,140]. This method has been applied for sugar reduction in cheese and yoghurt, where lactose was removed from the milk before processing into cheese and yoghurt [124,125,126,127]. 

During lactose hydrolysis by an enzyme, β-galactosidase, the galacto-oligosaccharides (GOS) formation occurs with a yield that is only around 40%. This suggests that a significant portion of the lactose remains unreacted, while some are converted into monosaccharides. Hence, the resulting raw GOS (rGOS) is a mixture of lactose, glucose, and galactose [141,142,143]. Galacto-oligosaccharides (GOS) are short oligosaccharides chains with several galactoses and one terminal glucose. In addition, it is observed that the lactose prevents the usage of GOS in the formulation of lactose-free foods for lactose intolerant. Furthermore, lactose along with glucose and galactose, increase the caloric value, while decreasing the prebiotic potential of GOS, thereby limiting its application to produce low-calorie foods. Therefore, removal of GOS by nanofiltration can be achieved to produce low calorie or low sugar foods for infants and diabetics [144,145]. Santibáñez et al. [146] were successful in removing monosaccharides and lactose with improved GOS retention using a hydrolysed rGOS nanofiltration technique with the TriSep XN45 membrane at 20 bar, 45 °C, and 1500 rpm.

### 5.3. Product Reformulation by Partial or Total Replacement with Sweeteners

Product reformulation by partial or total replacement with sweeteners is the most commonly used method as consumers prefer the sweet taste, and this method is suitable in various food matrices ranging from a solid to liquid foods [111]. Currently, there are several NSs (Table 1) and NNSs (Table 2) available for sugar substitution. However, the point to be noted is that the type of sweetener used for sugar substitution is product specific and no single sweetener and matrix model can be generally applied to any product [32]. The relative sweetness is approximate and varies with the type and concentration of the sweeteners. The relative sweetness of sweeteners is evaluated using the magnitude estimation method [147] considering a direct quantitative measurement of the subjective intensity of sweetness with a reference sucrose sample. The psychophysical relationship provides practical formulation information on increased sweetness intensity perception as a function of concentration [148]. Therefore, the concentration of a specific sweetener can only be determined by several trial and error experiments in the laboratory for individual products to check the desired sweetness achieved without much impact on consumer acceptance.

NNSs can be used for partial or total replacement of sugar in foods, however, there still exists common issues of differences in the temporal sensory profile [149] and bitter aftertastes [150,151]. However, the use of binary and ternary mixtures of sweeteners can overcome this issue to some extent [152,153]. Li, Lopetcharat, and Drake [42] were successful in partially reducing sugar in chocolate milk with stevia and monk fruit extract and maintaining a temporal sweetness profile. In line with this, the metallic aftertaste of Reb A (stevia compound) can be possibly masked by using a specific compound of monk fruit such as mogroside V40/V50 at different concentrations depending upon the product.

### 5.4. Gradual Reduction of Sugar

Gradual reduction is the method where sugar is slowly and progressively cut from the products, so that the consumers cannot easily distinguish the differences and gradually adapts to a lower sugar content without impacting their sensory recognition [110]. The threshold testing called a “just noticeable difference” (JND) determines the change in sugar concentration which causes the perceivable change in sweetness intensity by 50% of consumers [154]. This JND could be a valuable option to be explored for gradual sugar reduction without consumer awareness [119,155]. This strategy has already been successfully implemented in the UK for salt reduction in products containing high salt [156]. Organizations such as “Action on Sugar” [110] have suggested a similar strategy as the salt reduction program in the UK for sugar reduction in foods. However, the reduction in sugar over time might well be different from the slow reduction of salt. In fact, the JND has been implemented for sugar reduction in dairy-based emulsions and chocolate milk [129,157]. Hoppert et al. [157] investigated a matrix-specific sugar reduction and found that an individual was more sensitive to sugar reduction in products with higher fat concentration, i.e., the JND was low. On the basis of this model, 5 to 20% of sugar can be gradually reduced and the reduced sugar product still may be liked by the consumers although they may notice a difference in the sweetness in dairy-based emulsions [157] and chocolate milk [129]. Taking this into consideration, Oliveira et al. [129] reduced up to 12.9% of sucrose in chocolate milk without influencing liking by consumers. Furthermore, Li et al. [119] stated that a gradual sugar reduction under 30% had no significant influence on consumer acceptance. 

### 5.5. Use of Multisensory Interactions

The multisensory method is a technique where sugar reduction is achieved without the use of NNSs or any other sweeteners. It enhances the sensation perception by the aroma, colour, and other stimuli to perceive sweetness intensity [62]. The use of the aroma attribute could be a practical and viable alternative for sugar reduction, however, not as effective, in terms of magnitude, as with NNS approaches. Alcaire et al. [132] was able to reduce the effect of a 20% reduction of added sugar in milk desserts using aroma-related cross-modal interactions. Aroma is associated with the perception of sweetness in any specific product [158] and the taste-smell integration in the brain is related to existing experiences with taste-smell combinations. Tastants and odourants have both been revealed to generate overlapping activations in a specific part of the brain [159], thereby leading to enhanced sweetness perception. Contrary to this, the effect of colour on the perception of sweetness intensity is still unclear and leads to various interpretations. The enhanced perception of sweetness intensity because of a change in colour could be due to existing product experiences, as in the case of aroma. Spence et al. [160] demonstrated that the effect of colour on flavour and taste intensities was ambiguous. Therefore, the application of colour for sugar reduction is very limited, but still could be effective for specific product-colour combinations [62]. 

### 5.6. Heterogeneous Distribution

This is another unique method for sugar reduction which uses stimulation of taste receptors, serum release, as well as particle size and viscosity of foods to enhance the sweetness in foods [62]. The taste receptors play a vital role in the perception of sweetness intensity. Burseg et al. [161] showed that an ”on-off” mode of tastant had an increased perception of the tastant as compared with a constant-rate delivery of the tastant, which is known as pulsated delivery. Another possible way to enhance sweetness perception and reduce sugar in solid foods is by modifying the serum or fluid release from solid food matrices. In addition, particle-size and viscosity also play an important role in sweetness perception, but variably in solid and liquid foods [62].

## 6. Recent Trends in the Use of Natural Sweeteners for Sugar Reduction Strategies

Although artificial NNSs have been used in dairy-based foods and beverages, consumers prefer and demand products with natural sweeteners [162,163]. Consumers choose “all-natural” labelled products assuming them to be healthier, even without the knowledge of actual nutritional information displayed on the package [164,165,166,167]. For example, Li, Lopetcharat, and Drake [163] found that the parents preferred to buy the chocolate flavoured milk added with natural NNSs or sucrose over the artificial NNSs for their children. Similarly, Li, Lopetcharat, and Drake [42] and Oltman et al. [168] revealed the consumers’ preference of “naturally sweetened” labels for chocolate milk and protein beverages. Therefore, the use of natural NNSs such as stevia and monk fruit could provide better opportunities for consumer acceptance. 

### 6.1. Stevia (Stevia rebaudiana)

Stevia (*Stevia rebaudiana*) belonging to the Asteraceae family is native to Paraguay. However, today it is widely known and cultivated all over the world including some parts of Asia and Europe [169,170]. It is one of the natural low-calorie sweeteners commonly used in dairy products such as yoghurt and ice cream, even in baked goods and soft drinks [171,172,173]. The sweetness of stevia comes from the steviol glycosides present in stevia leaves. 

There are several compounds of steviol glycosides such as Rebusoside, steviolbioside, Stevioside, Rebaudioside A (Reb A), Reb B, Reb D, Reb E, Reb M, etc. depending upon the groups present at R1 and R2 positions (Table 4). Among these stevia compounds, Reb A is found in maximum proportion in the stevia leaf. When stevia was compared with other artificial NNSs (aspartame, sucralose, and neotame) in prebiotic chocolate dairy desserts for relative sweetness, it showed that neotame had the highest sweetening potency as compared with 8% sucrose (in the dessert), followed by sucralose, aspartame, and stevia [45]. In addition, Reb A has a lingering bitter or liquorice-like aftertaste. However, this can be masked by using other compounds such as Reb D and Reb M which are more similar to sucrose and do not have a bitter aftertaste [174]. These have the potential to replace greater proportions of sugar within foods/beverages, even without the use of taste modulators. Reb M has a more sucrose-like sensory profile as compared with Reb A. Reb M has faster sweetness onset, and lower bitterness lingering, sourness, and astringency than Reb A [174]. Reb M is produced by the enzymatic bioconversion of purified stevia leaf extract. Furthermore, Reb M has been approved in Canada for use as a food additive. It is intended to be used in food and beverages for human consumption in Australia and New Zealand at the permitted levels for steviol glycosides which is 4 mg kg^−1^ body weight day^−1^ [175]. Other compounds of steviol glycosides are still under the process of approval in different countries for their use in foods and beverages.

Additionally, some studies have revealed that stevia has possible hypotensive roles [176], in addition to increasing insulin sensitivity and glucose tolerance in humans [20]. Several in vitro and in vivo studies have suggested that stevia could be used to control glucose metabolism in diabetes. However, the mechanisms underlying the antidiabetic action have not been fully revealed and further in-depth research was required [177,178]. Furthermore, studies in animal models have revealed that NNSs (e.g., sucralose, saccharin, aspartame, acesulfame potassium, neotame, stevia, and monk fruit) interacted with sweet taste receptors expressed in enteroendocrine cells, and thereby increased the intestinal glucose absorption through the enhanced expression of Na-dependent glucose transporter isoform1 [179,180,181] and the movement of glucose transporter 2 (GLUT2) to the upper membrane of intestinal epithelial [182]. In contrast, studies conducted in people have shown mixed results for the effect of NNSs on plasma glucose and insulin regulation [183,184,185,186,187,188,189]. However, chronic effects on glucose metabolism could result from regular consumption of NNSs [179,180,181,190]. 

In addition, stevia leaf extract has also been reported to possess some therapeutic action due to antioxidant activity. Antioxidants scavenge the free radical electrons and superoxides and prevent damage to the tissues [191]. Shukla et al. [192] reported the in vitro potential of ethanolic leaf extract of stevia to be used as a natural antioxidant. The use of synthetic antioxidants such as butylated hydroxyanisole (BHA) and butylated hydroxytoluene (BHT) are posing a risk to several health hazards, therefore, there is a growing trend to replace these with the use of antioxidants from natural sources [193]. Furthermore, stevia also exhibits some antimicrobial activities and can inhibit the growth of infectious bacteria such as *Salmonella typhi*, *Aeromonas hydrophila, Vibrio cholerae, Bacillus subtilis,* and *Staphylococcus aureus* [194,195,196,197,198]. Steviol glycosides are considered to be safe, however, consumption that is more than the acceptable daily intake (ADI) limit of 4 mg kg^−1^ body weight day^−1^ may change the composition of the gut microbiota (EU regulation 1129/2011) [199,200,201].

### 6.2. Monk Fruit (Siraitia grosvenorii)

Another natural NNS among the trends for food application is the extract of monk fruit. Among the various compounds present in monk fruit, the mogroside V, which belongs to a family of triterpenoids, is the major one responsible for sweetness. It is derived from ripe monk fruit (*Siraitia grosvenorii*) also known as Luo Han Guo [43,150]. The fruit was discovered and classified initially in the 1930s and belongs to the family Cucurbitaceae [204]. The use of monk fruit has been authorised in Canada as a sweetener in foods with a maximum limit of 0.8% (as mogroside V). Furthermore, the monk fruit extract can be used as a low cost, high-intensity natural sweetener in various products [205]. The ADI of monk fruit has not been established, since no adverse effects have been reported, however, the ADI of monk fruit juice concentrate is approximately 25 mg kg^−1^ body weight day^−1^ [150].

Furthermore, this fruit has been used as a natural sweetener [206] and as a traditional medicine for the treatment of pharyngitis, pharyngeal pain, cough, cold, sore throat, constipation, and dire thirst in China [207,208]. Mogroside in monk fruit has shown beneficial health effects against diabetes, malignant tumor, and inflammation in animal models [209,210] and could be used as a low-calorie sweetener for diabetic patients [206]. The extract of mogroside is effective in the oxidative modification of low-density lipoprotein [211]. The in vitro results for antioxidant activity revealed mogroside V to have reactive oxygen species (ROS) scavenging ability [206]. Similarly, Lim [212] reported the antioxidant activity of monk fruit extracts and its potential role for anticancer, antiviral, antihyperglycemic, and antidiabetic activities. 

## 7. Sweeteners Used for Sugar Reduction in Chocolate Flavoured Milk

Several researchers have tried to reduce sugar in chocolate flavoured milk using different strategies and sweeteners (Table 5). Rad et al. [213] evaluated the effect of stevia on the physical properties of chocolate milk and found that above 50% substitution by stevia had a negative impact on sedimentation and viscosity (Table 5). Similarly, Li, Lopetcharat, and Drake [42] considered consumer acceptance for the optimisation of Monk fruit extract and stevia leaf extract separately in skim chocolate milk and found a partial reduction of sucrose with substitution by monk fruit extract or stevia leaf extract to have a sensory profile comparable to control milk. Alternatively, Zhang and Gruen [214] estimated the iso-sweetness of rebaudioside A (Reb A), monk fruit, erythritol, lactitol, and xylitol with respect to 10.1% sucrose-sweetened whey protein beverages and found Reb A to have the highest sweetness potency as compared with others. Bordi Jr et al. [215] was successful in reducing 35% of sugar in chocolate milk using 150 ppm of Reb A stevia without impacting overall liking. Furthermore, Azami et al. [216] used liquorice extract as a sugar substitute in chocolate milk and studied the microbial aspect, as well as consumer acceptance and physicochemical properties, where no significant variations in acidity, pH and microbial growth were seen, however, higher colour and sedimentation stability as compared with the control were observed. These studies suggested that a considerable amount of added sugar could be reduced in chocolate flavoured milk using an appropriate strategy and sweeteners (which was product specific) without compromising the sensory properties, physicochemical properties, or microbial safety. However, the limitation was that most studies focused on consumer acceptability but not on the changes in the stability and physicochemical properties.

## 8. Consumer Preference for Flavoured Milk and Possible Solutions for Sugar Reduction

Consumers’ preferences are continuously shifting towards healthier food alternatives. This is mainly due to the increasing awareness in consumers regarding the impact of foods on health [218]. Most of the dairy and other beverages’ companies have focused on producing healthier products without compromising changes in the sensory profile to maintain the market value, since a better sensory profile of the product is vital for consumer liking and acceptance of the product [219]. Furthermore, consumer acceptance of chocolate flavoured milk is largely dependent on the sweetness and texture profile [220]. Texture profile is further dependent on ingredients such as sugar, fat, protein, as well as stabilisers like carrageenan, and other thickening agents present in chocolate milk [221]. Overall, it seems that there is not a single driver, but multiple drivers including flavour, sweetness, and mouthfeel for the consumer acceptance of chocolate milk which also holds for other beverages. These drivers will vary with the variation in sugar concentrations. In fact, sugar as well as other components (e.g., fat, protein, salt, stabiliser, flavour, etc.) present in a food matrix work in synergy rather than individually to maintain an optimum food matrix and provide nutrition and health effects [222]. The fat and other food components may be important factors for influencing the intensity and liking of sweetness and consumption of sugar in beverages [223]. However, there is limited information on the interaction of different food components and their influence on taste and consumer acceptance. Therefore, while reducing sugar, it is critical to analyze how product properties (physicochemical and microbial) vary with sugar concentrations and how they influence the consumer preference and acceptance of the product. 

The reduction of sugar and its impact on consumer acceptance can be overcome using sweeteners to enhance the sensory and functional properties of sucrose to some extent. No single sweetener has similar functionality to sucrose, therefore, the use of two or more sweeteners as a blend can provide a flavour and taste profile similar to that that of sucrose. For example, the combined effect of stevia and sucralose has improved sensory and physical properties in the sugar-free dairy dessert [44]. In addition, the use of two sweeteners as a blend (Cyclamate/Saccharin blend, 2:1) minimises off-flavour or bitter aftertaste in peach nectar [224]. Similarly, blending Reb M with Reb B/Reb D resulted in sweetness synergy with an improvement in sweetness intensity, onset and bitterness perception [174]. Similarly, blending aspartame (APM) and acesulfame-K (ACE-K) resulted in sweetness synergy by approximately 30% [150]. Furthermore, aroma plays a significant role in taste perception [225]. It can either mask or increase the perception of a taste [226]. As sweetness perception is enhanced by the addition of vanilla, caramel, or fruity aromas [63,64,65], their usage enhances the sensory perception of products. Therefore, the best possible solution could be to use a holistic approach utilizing various sugar reduction strategies along with the use of natural NNSs in trends such as stevia and monk fruit compounds with a superior sensory contribution. 

## 9. Sugar and Energy Content of Commercial Chocolate Flavoured Milk

Flavoured milk consumption is popular among both adults and children. It provides essential nutrients similar to plain milk (with 4–5% sugar) but with added sugar and flavour in varying amounts [227]. Chocolate milk is the most popular and frequently consumed product among all other flavoured milk [216,228]. Normal commercial chocolate milk contains 8–13% total sugar (Table 6, the ones with the lower values are with partially reduced sugar, reduced fat, or lactose-free) of which half of the sugar comes from naturally occurring lactose in milk and the remainder from added sugars. The consumption of chocolate flavoured milk helps to meet the recommended daily intake for dairy products and some nutrients such as calcium and potassium [229,230] but the extra added sugar leads to additional calories (1 g sugar = 4 calories and 1 calorie = 4.2 joules) as summarized in Table 6. This is further linked to the occurrence of overweight, obesity [2,3], and type 2 diabetes [4,5], as stated earlier. Therefore, reducing added sugar content in chocolate milk will help reduce the calorie contribution through its consumption. 

## 10. Future Prospective and Conclusions

The key findings of this review are that the sugar can be reduced by employing several strategies namely lactose hydrolysis, ultra- and nanofiltration, total/partial replacement of sugar, gradual reduction, multisensory interactions, or heterogeneous distribution. However, all of these methods have their advantages and disadvantages. The use of any method or sweetener is product specific and can vary with the type of product, both with solid and liquid food products. Furthermore, limitations with nutritional studies lie with the challenge to optimize the correct proportion of ingredients in a reformulated food product as all the individual ingredients have their specific function to perform. Some ingredients work in synergy, while some mask or inhibit the effect of others in a complex food matrix system. In addition, as no single sugar has a sensory profile similar to sucrose, therefore, trying a combination of two or more natural NNSs among trends could be an option for food industries to reduce sugar but still maintain consumer liking and acceptance of the product. However, their potential application in different food products and the impact on the sensory, physicochemical, and nutritional properties, in addition to food safety issues must be carried out. Furthermore, the use of multiple strategies outlined in this review could incredibly assist food companies to overcome the technical challenges underlying sugar reduction and to achieve at least a small reduction that could benefit the health of the population significantly.

## Figures and Tables

**Table 1:** Natural and artificial nutritive sweeteners (NSs), their advantages, disadvantages, sweetness potency, and calorie contribution.

Nutritive Sweeteners (NS)	Structure	Type	Advantages	Disadvantages	* Sweetness Potency	Calorie/g	References
Sucrose	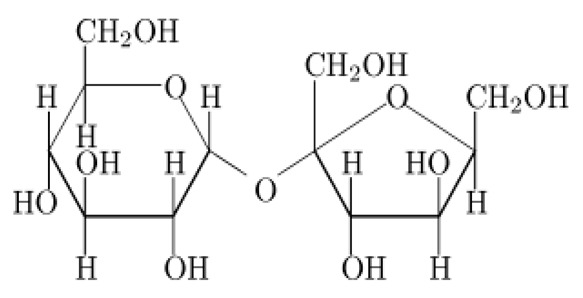	Natural	Provides colour, flavour, bulkness and preservative actions against microbes	Contributes calories to diets	1.0	4.0	[22]
Glucose	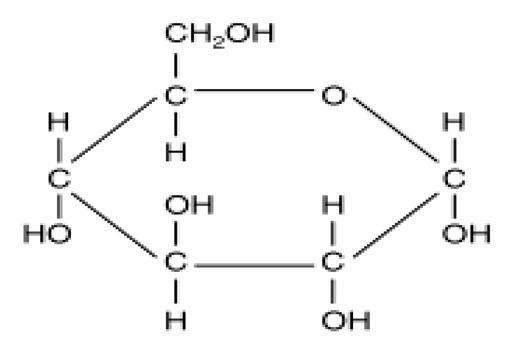	Natural	Essential energy source for the brain	Contributes calories to diets and affects satiety	0.75×	4.0	[23]
Fructose	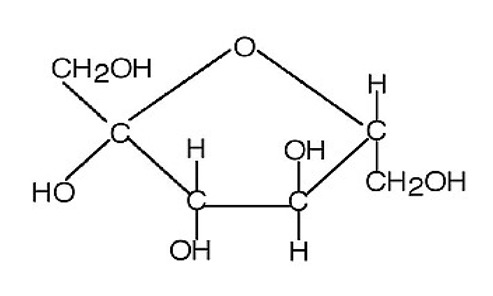	Natural	Sweetest carbohydrate in nature	Contributes calories to diets but does not affect satiety like glucose	1.5–1.8×	4.0	[24]
Lactose	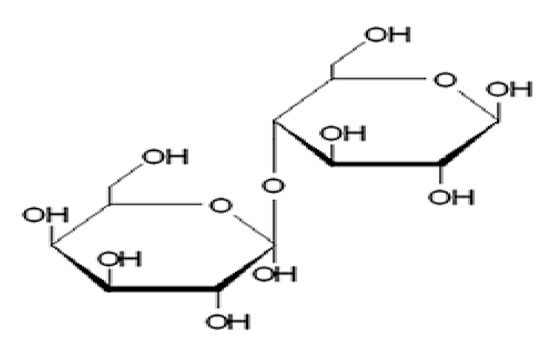	Natural	Raw material and prebiotics for probiotics	Less contribution to sweetness	0.11–0.13×	3.9	[25,26]
Trehalose	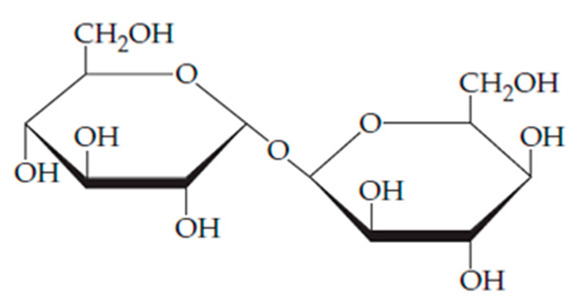	Artificial	Antioxidant activity, food flavour enhancer; prevents starch aging; odor reduction and extension of the shelf life	Contributes calories	0.5–0.7×	3.6	[27,28,29,30]
Erythritol	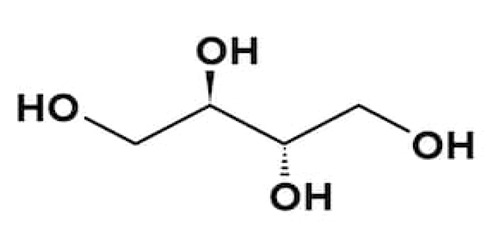	Artificial	Highly stable, low calorie contribution, tooth-friendly sweetener providing volume, texture, and microbiological stability	Can cause gastrointestinal symptoms	0.7×	0.2	[31]
Isomalt (Isomaltitol)	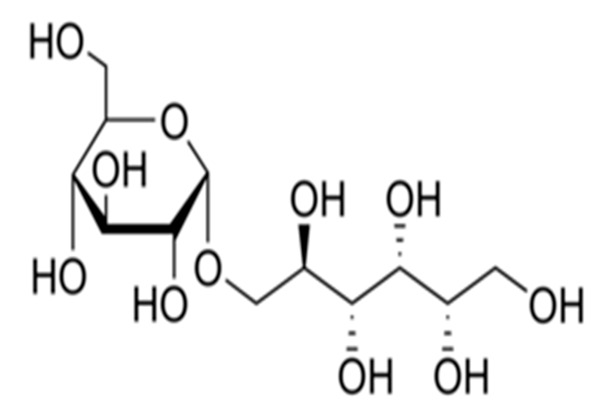	Artificial	Heat resistant and tooth-friendly	Laxative effect along with gastrointestinal symptoms (abdominal discomfort, bloating and flatulence if consumed in excess i.e., >50 g)	0.45–0.6×	2.0	[31,32]
Lactitol	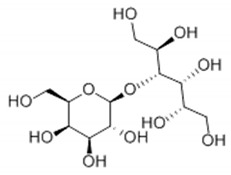	Artificial	Low calories suitable for sugar-free foods	Similar to Isomalt	0.35–0.4×	1.9	[31]
Maltitol	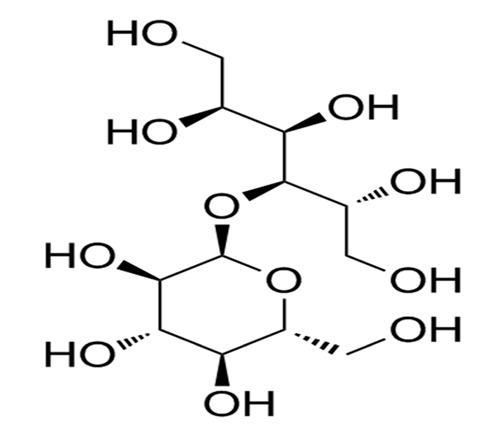	Artificial	Heat resistance, strong flavour consistency, and tooth-friendly as it is not fermented by tooth plaque forming microorganisms	Similar to Isomalt	0.5–0.9×	3.0	[31,33]
Sorbitol	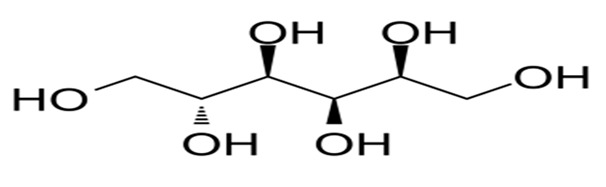	Artificial	Bulking agent, humectant, sequestrant and acts as stabilizer	Similar to Isomalt	0.6×	2.6	[31,34]
Mannitol	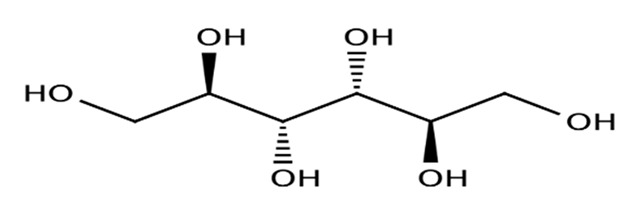	Artificial	Crystallization in the form of a colourless/white needle/rhombus with extremely low hygroscopicity	Only 18% (*w/v*) soluble in water at 25 °C	0.5–0.72×	1.6	[35,36,37]
Xylitol	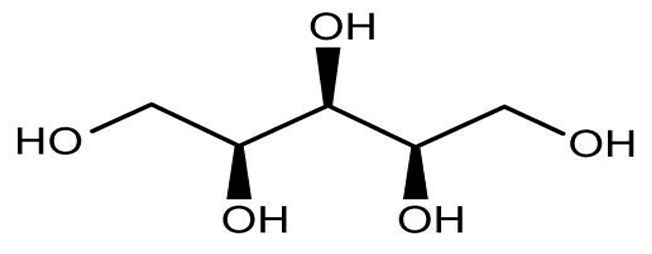	Artificial	Sweetness intensity similar to sucrose	Similar to Isomalt	1.0×	3.0	[32]

* Sweetness potency-the indicated estimate values are times (×) that of sucrose.

**Table 2:** Natural and artificial non-nutritive sweeteners (NNSs) used for sugar reduction in dairy products.

Non-nutritive Sweetener (NNS)	Structure	ADI (mg/kg Body Weight/day)	Onset	Lingering	Off-taste	Food and Beverages	Amount of Sugar Reduction	Reference
**Natural**								
Thaumatin	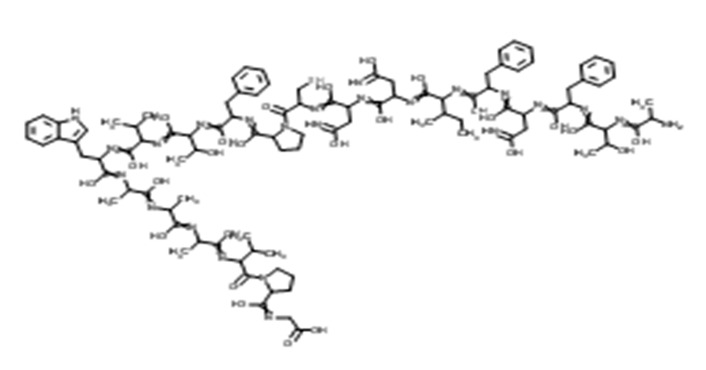	50	Delay	Long	Nil	Probiotic chocolate-flavoured milk	20%	[15,38,39]
Neohesperidine dihydrochalcone	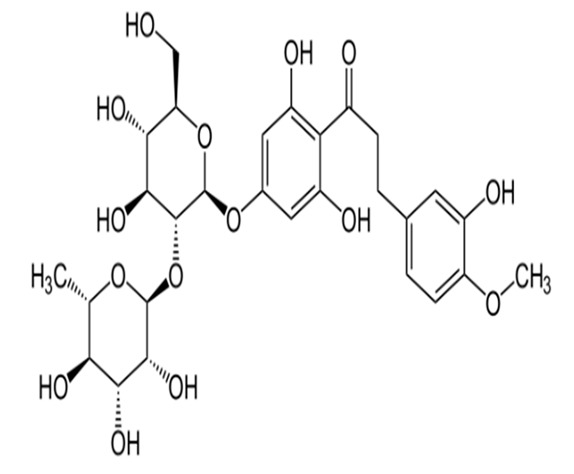	35	Delay	Long	Like licorice	Chocolate, skimmed plain yoghurt	-	[15,40,41]
Steviol glucosides	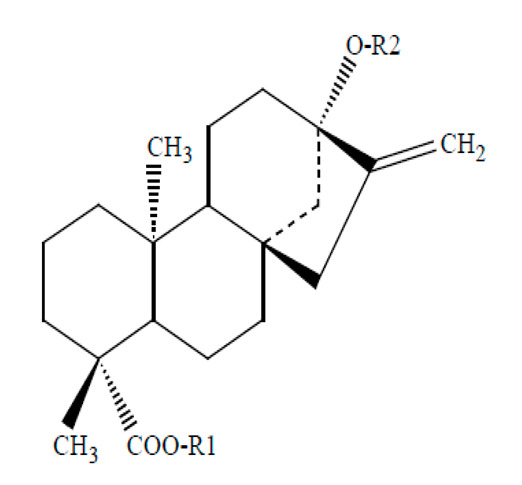	4	Delay	Moderate	Bitter	Chocolate milk, chocolate dairy desserts	50%	[15,42,43,44,45]
Monk fruit (Mogrosides V)	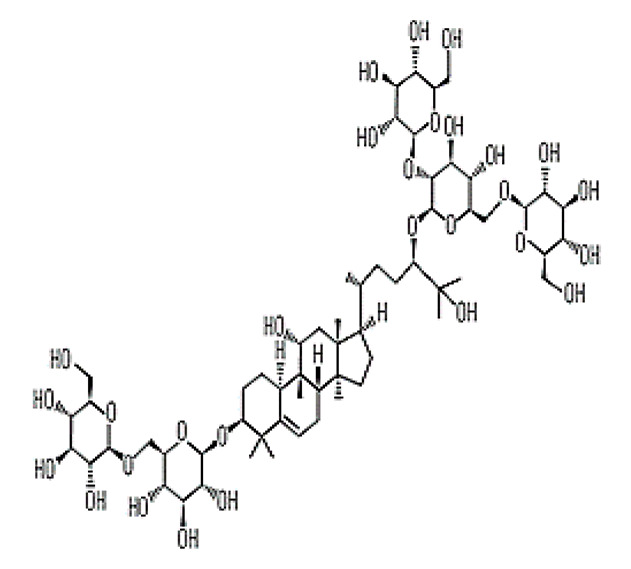	25	Delay	Long	Nil	Chocolate milk	50%	[42,43,46]
**Artificial**								
Advantame	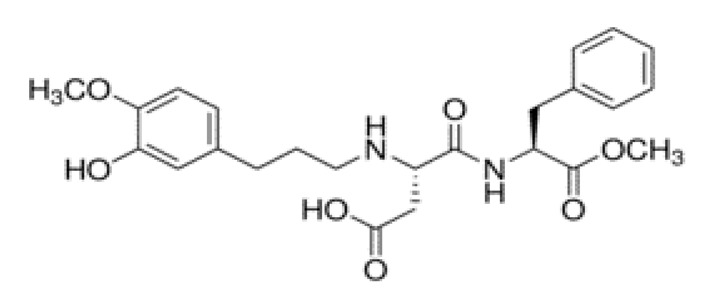	5	Delay	Moderate	Nil	Strawberry-flavoured yoghurt	100%	[15,47,48]
Neotame	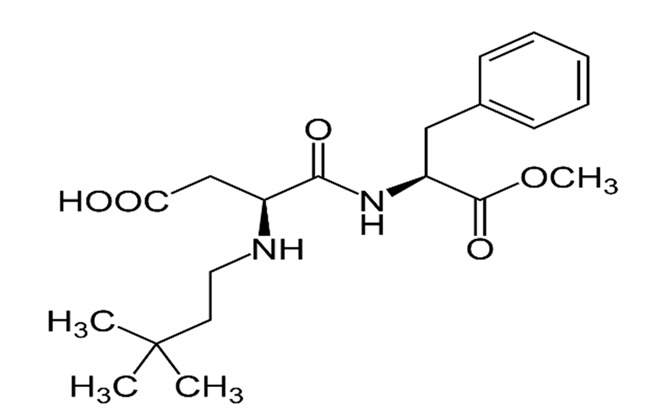	2	Delay	Strong	Nil	Prebiotic chocolate dairy dessert	100%	[15,45]
Sucralose	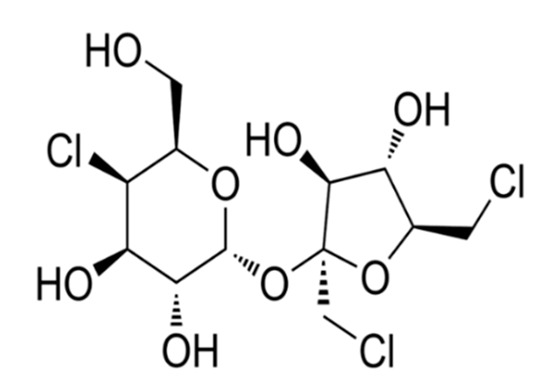	5	Slight delay	Moderate	Slight bitter	Strawberry flavoured yoghurt, dairy desserts, lassi	100%	[15,44,48]
Saccharin	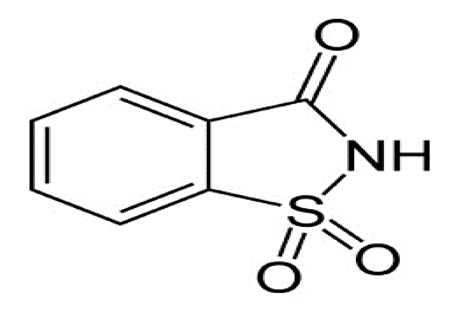	5	Rapid	Non-significant	Bitter and metallic	Strawberry flavoured yoghurt, lemon whey beverages	39%-100%	[15,48,49]
Aspartame	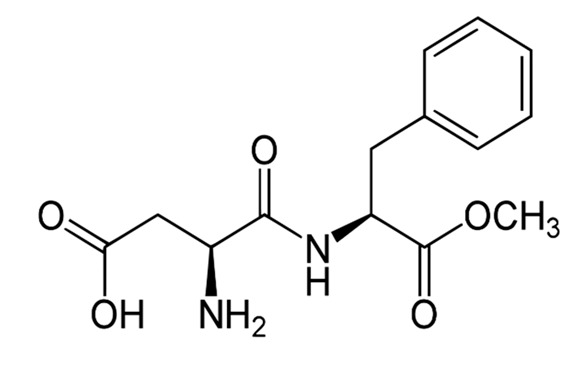	40	Slight delay	Moderate	Non-significant	Strawberry flavoured yoghurt, lemon whey beverages, lassi	39–100%	[15,49,50]
Acesulfame K	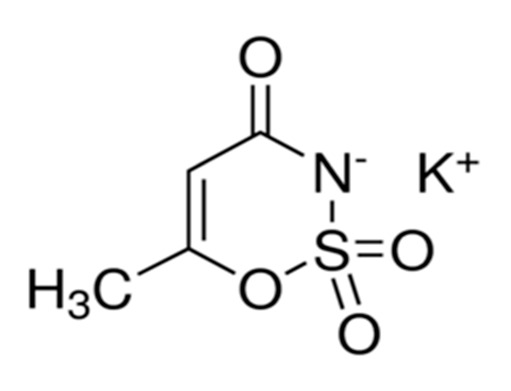	9	Quick	Low	Bitter and metallic	Strawberry flavoured yoghurt, lassi	100%	[15,50]
Cyclamates	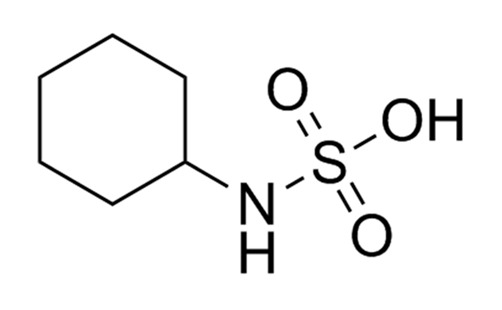	11	Rapid	Non-significant	Bitter and salty	Strawberry flavoured yoghurt	100%	[15]

**Table 3:** Sugar reduction strategies, feasibility, and their applications in milk-based products and other foods.

Method	Advantage	Disadvantage	Use of Sweeteners	Application	Example	Reference
Lactose hydrolysis	Natural and easily achievable process	Quite expensive and desired sweetness might not be achieved	Yes/No	Lactose hydrolysis applicable in milk only, though the hydrolysed milk can be used to make milk-based products	Flavoured milk, yoghurt, and ice-cream	[119,120,121,122,123]
Ultra-/nanofiltration	Relatively easy, quick, and cost-effective process	Works better in conjunction with lactose hydrolysis process	Yes/No	Applicable in lactose hydrolysed milk which then can be used to make milk-based products	Cheese and yoghurt	[124,125,126,127]
Product reformulation (partial and total replacement)	Substantial amount of sugar can be reduced	Sensory profile and satiety value of sucrose cannot be replaced totally	Yes	The most common approach in both solid and liquid foods	Probiotic/chocolate flavoured milk, jam, chocolate, juice	[39,42,128]
Gradual reduction	Relatively easy process	Consumers should accept the changed sensory profile	No	Both in solid and liquid foods	Chocolate flavoured milk, salt	[129,130]
Multisensory interactions	Formulation easy and achievable without sweeteners	High level of sugar reduction cannot be achieved	No	Both in solid and liquid foods (aroma); liquid foods (colour)	Milk desserts, cheese, orange juice, strawberry yoghurt, vanilla milk desserts	[128,131,132,133]
Heterogenous distribution	The composition of the product is minimally affected	Achievable only on small scale	No	Solid foods (stimulation of taste receptors, serum release, reducing particle-size) and liquid foods (reduced viscosity)	Semi-solid food gels, beverages, dairy desserts	[134,135,136,137,138]

**Table 4:** Different compounds of steviol glycosides with varying groups at R1 and R2 positions [174,202,203].

Compound	R1	R2	Chemical Formula	Structure	* Sweetness Potency
Rubusoside	Glucose β1 (Glcβ1-)	Glcβ1-	C_32_H_50_O_13_	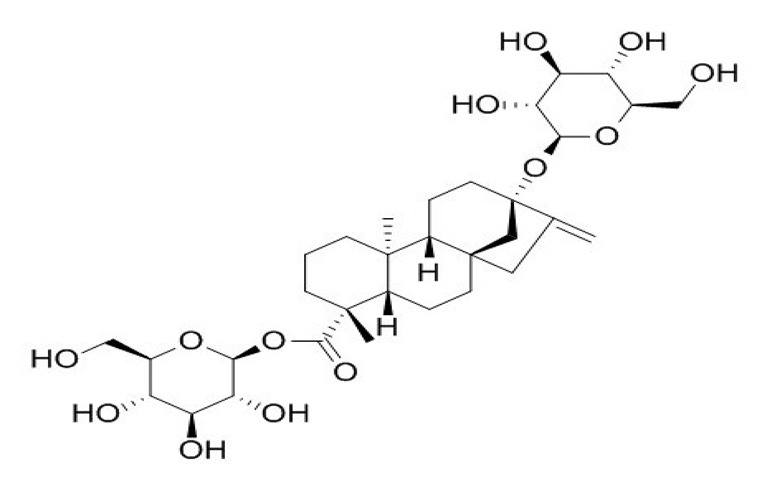	114
Steviolbioside	H	Glcβ(1-2)Glcβ1-	C_32_H_50_O_13_	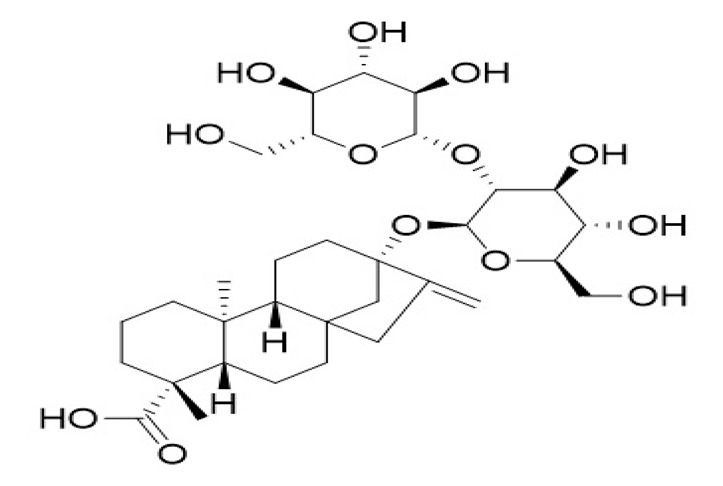	90
Stevioside	Glcβ1-	Glcβ(1-2)Glcβ1-	C_38_H_60_O_18_	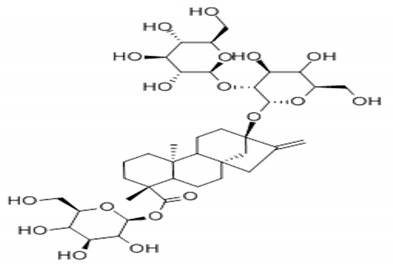	210
Rebaudioside B (Reb B)	H	Glcβ(1-2)[Glcβ(1-3)]4.2.4Glcβ1-	C_38_H_60_O_18_	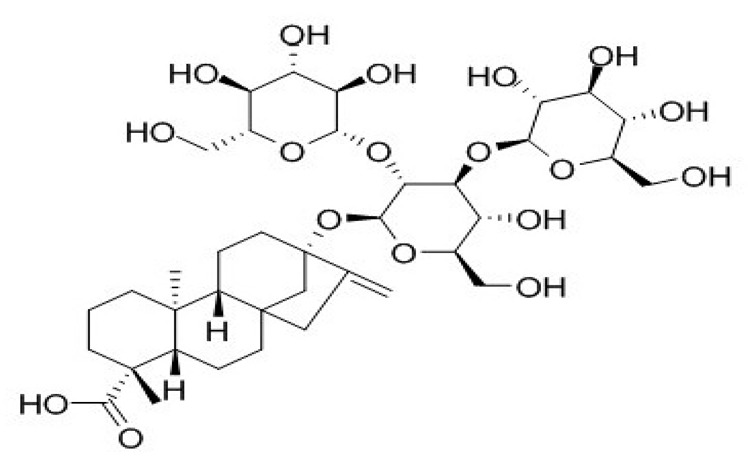	150
Reb E	Glcβ(1-2)Glcβ1-	Glcβ(1-2)Glcβ1-	C_44_H_70_O_23_	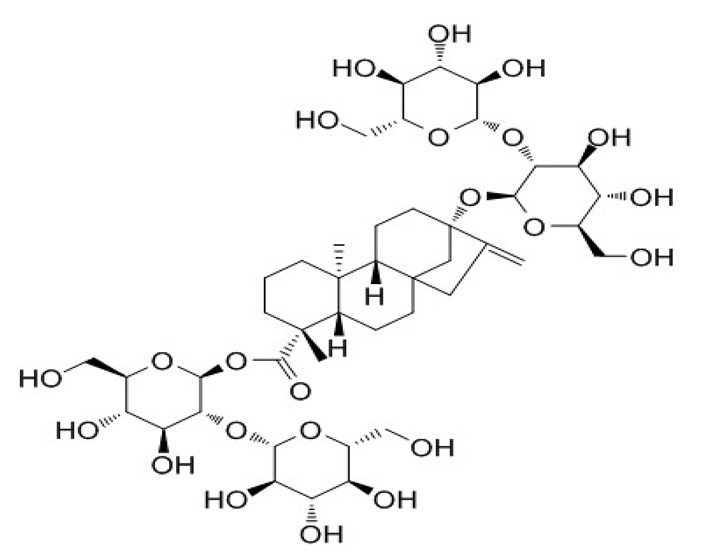	174
Reb A	Glcβ1-	Glcβ(1-2)[Glcβ(1-3)]Glcβ1-	C_44_H_70_O_23_	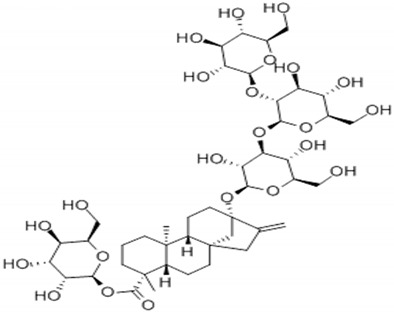	200
Reb D	Glcβ(1-2)Glcβ1-	Glcβ(1-2)[Glcβ(1-3)]Glcβ1-	C_50_H_80_O_28_	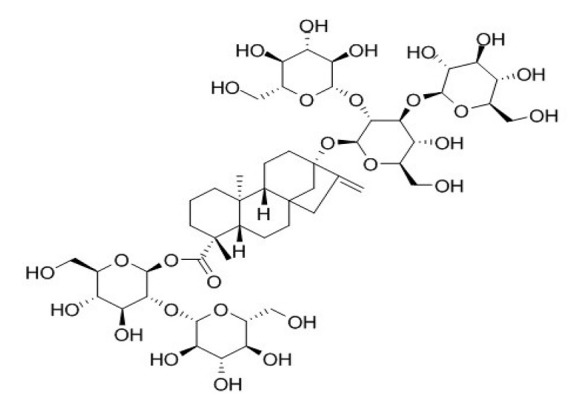	221
Reb M	Glcβ(1-2)[Glcβ(1-3)]Glcβ1-	Glcβ(1-2)[Glcβ(1-3)]Glcβ1-	C_56_H_90_O_33_	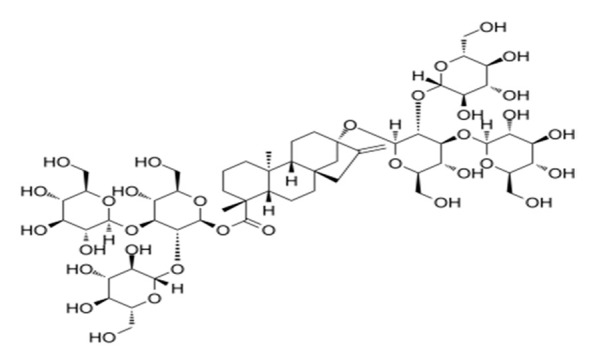	250
Dulcoside A	Glcβ1-	Rhamnose (Rha) Rhaα(1-2)Glcβ1	C_38_H_60_O_17_	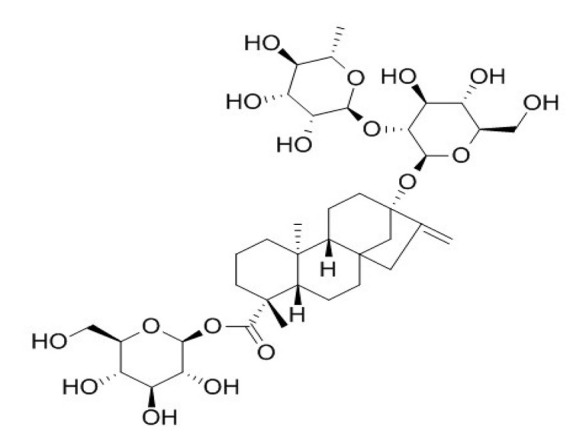	30
Reb C	Glcβ1-	Rhaα(1-2)[Glcβ(1-3)]Glcβ1-	C_44_H_70_O_22_	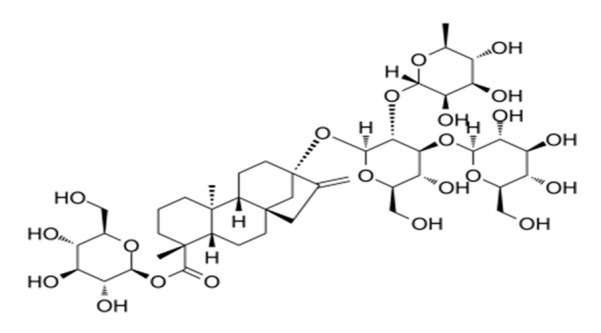	30
Reb N	Rhaα(1-2)[Glcβ(1-3)]Glcβ1-	Glcβ(1-2)[Glcβ(1-3)]Glcβ1-	C_56_H_90_O_32_	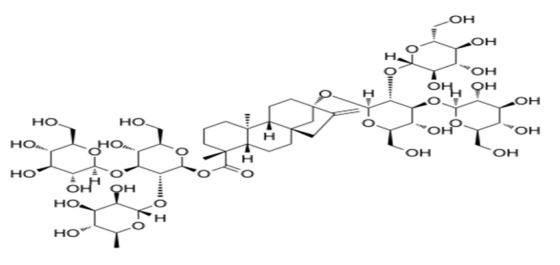	-
Reb O	Glcβ(1-3)Rhaα(1-2)[Glcβ(1-3)]Glcβ1-	Glcβ(1-2)[Glcβ(1-3)]Glcβ1-	C_62_H_100_O_37_	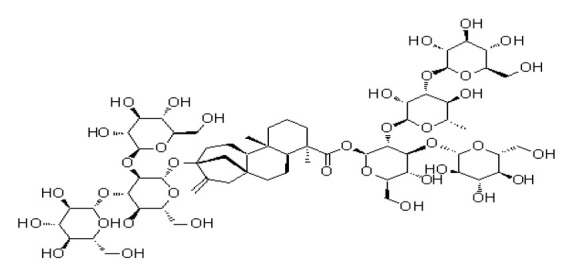	-
Reb F	Glcβ1-	Xylose (Xyl) Xylβ(1-2)[Glcβ(1-3)]Glcβ1	C_43_H_68_O_22_	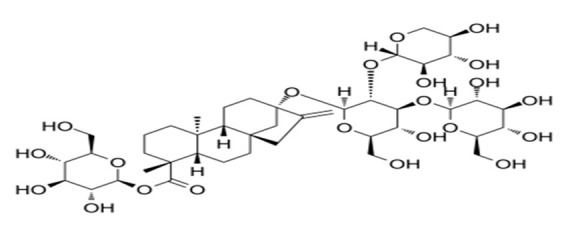	200

* Sweetness potency-the indicated estimate values are times that of sucrose.

**Table 5:** Different sweeteners and methods used for sugar reduction in chocolate flavoured milk.

Method	Sweetener/Others	Consumer Acceptance Study	Physicochemical Study	Microbial Study	Outcomes	Reference
Partial (50%)/total (100%) substitution	Stevia/inulin (thickening agent)	No	Sedimentation and viscosity	No	100% stevia increased precipitation, decreased viscosity while 50% stevia with inulin had better physical property	[213]
Partial substitution	Monk fruit extract/stevia leaf extract	9-Point hedonic, just about right (JAR)	No	No	Sucrose (39.7 g/L) with monk fruit extract (46 mg/L) or stevia leaf extract (30 mg/L) had sensory profile comparable to control milk (51.4 g/L sucrose)	[42]
Partial substitution	Thaumatin/vanilla (probiotic culture)	9-Point hedonic, temporal check-all-that-apply (TCATA)	No	No	20% sugar reduction as compared with a control (9% sugar) affected sweetness perception but not overall liking; vanilla increased sweetness perception only with 40–60% sugar reduction; 10 ppm of thaumatin showed increased sweetness perception only when sugar reduction was 60%	[39]
Partial (50%)/total (100%) substitution	D-tagatose/probiotic strains	5-Point hedonic scale	pH, redox potential, acidity	No (probiotic strains evaluated)	Probiotic strains and D-tagatose significantly affected probiotic viability, physical and chemical properties of chocolate milk. Therefore, proper selection of sugar ratio is recommended	[217]
Partial substitution	Reb A stevia	Preference test (7-point hedonic)	No	No	150 ppm of stevia was optimum for 35% sugar reduction without difference in overall liking	[215]
Gradual reduction	-	9-Point hedonic, CATA	No	No	12.9% of sugar can be reduced by a fraction of 6.66% added sugar in two sequent reductions to maintain consumer liking	[129]
Partial substitution	Liquorice extract powder (LEP)/cocoa powder (CP)	5-Point hedonic scale, preference ranking test	Sedimentation, pH, acidity, colour	Total bacterial and yeast count	0.35:0.65; LEP/CP (based on 1/100 g CP) and 5 g per 100 g sucrose was optimum for consumer acceptance with no significant variations in acidity, pH, and microbial growth but significantly higher colour and sedimentation stability than the control	[216]

**Table 6:** Composition of Australian commercial chocolate flavoured milk.

S.N.	Chocolate Flavoured Milk	Manufacturer	Energy (KJ)	Fat (%)	Sugar (%)	Protein (%)	Salt (Na, %)
1	Big M original	Part of LD&D Australia PTY LTD	284	1.80	9.50	3.20	0.04
2	Norco fm	North Coast Fresh Food & Cold Storage Co-operative Company Ltd.	327	1.90	11.00	4.00	0.05
3	Norco NATURAL Malt, Honey and Chocolate	North Coast Fresh Food & Cold Storage Co-operative Company Ltd.	423	3.70	10.60	4.20	0.08
4	Norco REAL Iced Chocolate	North Coast Fresh Food & Cold Storage Co-operative Company Ltd.	411	3.90	10.80	4.20	0.05
5	Norco REAL FUEL	North Coast Fresh Food & Cold Storage Co-operative Company Ltd.	422	3.60	9.30	6.00	0.05
6	RAM	Farmdale	360	3.50	8.30	3.70	0.05
7	Coach House Dairy	NuLac Foods P/L, Australia	551	8.00	11.00	4.00	0.04
8	OAK Chocolate	Parmalat Food Products PTY LTD	376	3.40	10.60	3.50	0.05
9	UP & GO Liquid Breakfast	Sanitarium (Australia Health & Nutrition Assoc. Limited)	327	1.50	7.70	3.30	0.06
10	Barista Bros Butterscotch Brownie	Coca-cola Amitil (Australia) PTY LTD	226	1.40	7.10	2.70	0.06
11	EMMA & TOMS	EMMA & TOMS Foods PTY LTD	188	1.30	4.80	3.50	0.04
12	Pauls ZYMIL	Parmalat Australia PTY LTD	317	3.10	8.30	3.30	0.04
13	Pauls Farmhouse Gold	Parmalat Australia PTY LTD	419	5.00	9.80	3.60	0.04
14	Pauls Farmhouse Gold chocolate custard	Parmalat Australia PTY LTD	515	5.00	13.00	3.20	0.04
15	Big M Double Choc	Big M (Part of LD&D Australia PTY LTD)	302	2.50	8.90	3.30	0.04
16	OAK Chocolate	Parmalat Food Products PTY LTD	376	3.40	10.60	3.50	0.05
17	OAK THE MAX COOL CHOC MINT	Parmalat Food Products PTY LTD	373	3.40	10.30	3.70	0.05
18	OAK PLUS PROTEIN	Parmalat Food Products PTY LTD	299	1.40	7.80	6.00	0.06
19	OAK THICK CHOC MINT	Parmalat Food Products PTY LTD	383	3.50	10.90	3.60	0.05
20	OAK THICK DEATH BY CHOCOLATE	Parmalat Food Products PTY LTD	381	3.50	10.90	3.60	0.05
21	Norco Mighty Cool	NORCO (North Coast Fresh Food & Cold Storage Co-operative Company Ltd.)	263	1.40	8.20	3.90	0.06
22	RAM BERT	Farmdale	269	1.30	8.40	3.80	0.05
23	Woolworths Chocolate milk	Woolworths	235	1.40	5.80	3.90	0.04
24	Pauls MILKY MAX	Parmalat Australia PTY LTD	298	1.80	10.30	3.20	0.05
25	Dairy Farmers Fresh milk	Dairy Farmers	248	1.80	7.10	3.20	0.05
26	EDGE BIG M CHOCOLATE	Big M (part of LD&D Australia PTY LTD)	283	1.80	8.80	3.30	0.04
27	Nippy’s ICEDCHOCOLATE	KNISPEL BROS PTY LTD	267	1.70	7.90	3.00	0.04
28	Moo Chocolate	Devondale	334	3.40	9.10	3.00	0.05
29	Breaka Chocolate	Parmalat Australia PTY LTD	316	2.00	10.20	3.70	0.05
30	Since 1967 OAK Chocolate	Parmalat Food Products PTY LTD	360	2.00	12.20	3.60	0.06
31	Nestle Ready to Drink Chocolate	Nestle	300	1.40	8.40	4.00	0.05
32	M2GO	Alfred Foods	281	1.80	9.40	3.10	0.04
33	LIDDELLS Lactose free Chocolate Milk	LIDDELLS	313	3.30	7.90	3.00	0.03
34	LIDDELLS Lactose free, 99% Fat Free Chocolate Milk	LIDDELLS	263	1.00	9.80	3.20	0.07

Source: Information gathered from online and in-person supermarkets (Coles, Woolworths, ALDI, and IGA) survey near Burwood, Australia.
